# *Tol2*-Mediated Generation of a Transgenic Haplochromine Cichlid, *Astatotilapia burtoni*

**DOI:** 10.1371/journal.pone.0077647

**Published:** 2013-10-25

**Authors:** Scott A. Juntti, Caroline K. Hu, Russell D. Fernald

**Affiliations:** Department of Biology, Stanford University, Stanford, California, United States of America; National University of Singapore, Singapore

## Abstract

Cichlid fishes represent one of the most species-rich and rapid radiations of a vertebrate family. These ∼2200 species, predominantly found in the East African Great Lakes, exhibit dramatic differences in anatomy, physiology, and behavior. However, the genetic bases for this radiation, and for the control of their divergent traits, are unknown. A flood of genomic and transcriptomic data promises to suggest mechanisms underlying the diversity, but transgenic technology will be needed to rigorously test the hypotheses generated. Here we demonstrate the successful use of the *Tol2* transposon system to generate transgenic *Astatotilapia burtoni*, a haplochromine cichlid from Lake Tanganyika, carrying the *GFP* transgene under the control of the ubiquitous *EF1α* promoter. The transgene integrates into the genome, is successfully passed through the germline, and the widespread GFP expression pattern is stable across siblings and multiple generations. The stable inheritance and expression patterns indicate that the *Tol2* system can be applied to generate *A. burtoni* transgenic lines. Transgenesis has proven to be a powerful technology for manipulating genes and cells in other model organisms and we anticipate that transgenic *A. burtoni* and other cichlids will be used to test the mechanisms underlying behavior and speciation.

## Introduction

Cichlid fishes form the most species-rich vertebrate family known. Approximately 2200 species have been described, and they exhibit spectacular variation in morphology, physiology, and behavior [1], [2], [3]. The majority (∼2000 species) of cichlid species are found in the Rift Valley lakes of Africa and have undergone explosive radiation over the past 2–5 million years [4], [5], making the family Cichlidae an excellent model for studying speciation. Cichlid behavior has also been extensively studied because these species vary in social structure, parenting strategy, feeding method, and conspecific communication [6], [7], [8], [9], [10]. Thus, cichlids represent a trove of information valuable to scientists studying the bases of variation in these traits, and the molecular basis of speciation.

Recently, new molecular resources for cichlid biologists have become available, including whole genome sequences for five species (Broad Institute, Boston, MA, USA), tissue-specific transcriptomes, genetic linkage maps, and BAC libraries [11], [12], [13]. Three of the sequenced species (*Astatotilapia burtoni*, *Pundamilia nyererei*, and *Metriaclima zebra*) are in the haplochromine tribe, the cichlid lineage with the highest speciation rate and extensive niche diversity [5]. Haplochromines' rapid diversification has enabled investigators to hybridize species and perform quantitative trait loci (QTL) analysis [14], [15]. While new genomic data can greatly speed the discovery of loci that correlate with species-specific traits, functional testing of candidate genes or regulatory regions with genetic tests of necessity and sufficiency has not yet been performed in cichlids. Data from such tests using transgenesis would allow a formal test of the role of individual genes in the divergence of cichlid traits. For example, transgenic vectors could be used to deliver an allele from one species to another species bearing a predicted recessive mutation, permitting a test of gene function. Additionally, transgenesis permits the manipulation of cell populations by driving heterologous transgenes in a restricted pattern, enabling the analysis of the role of specific cell groups in developmental processes or neural circuit function [16].

**Figure 1:**
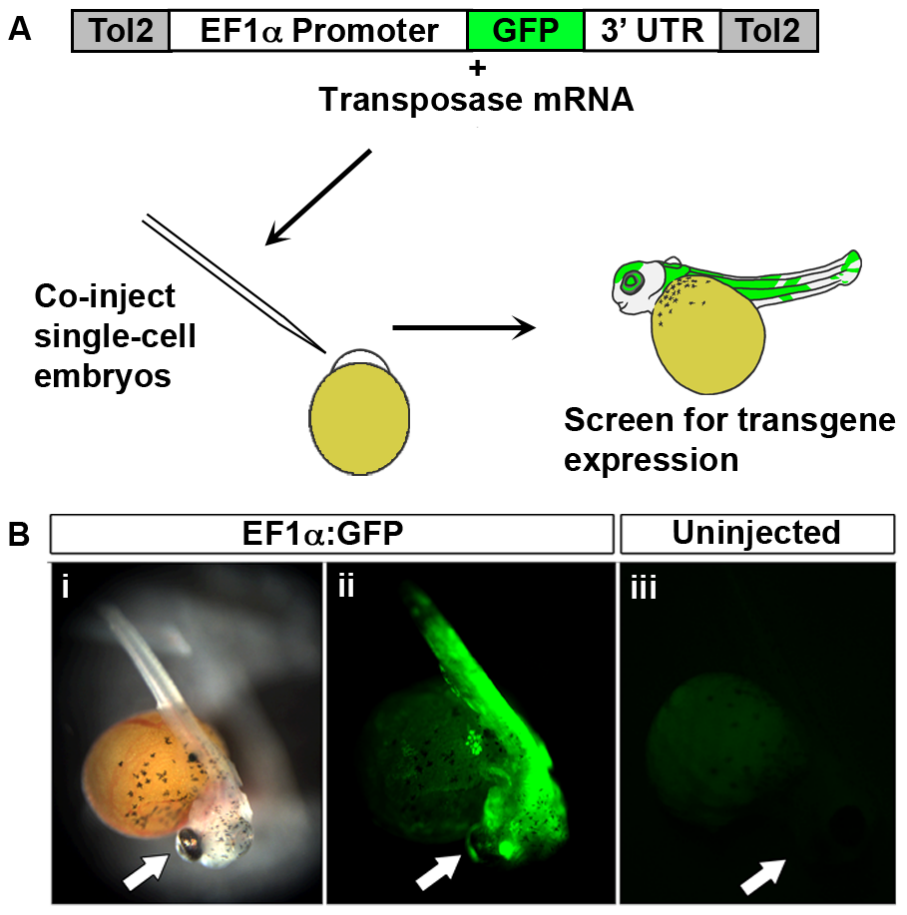
Production of transgenic *A. burtoni.* A) *Tol2*-flanked transgenic construct DNA (pT2KXIG
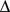
n) is co-injected with mRNA encoding transposase into fertilized single cell embryos. B) Images of embryos 6 days post-fertilization. i) Brightfield image of *EF1α*:*GFP*-injected embryo; ii) Robust GFP expression in embryo shown in i; iii) Uninjected embryo. Arrows mark the heads of embryos. Note that yolk exhibits autofluorescence.

Work using traditional model organisms has successfully utilized transposon-mediated approaches to insert transgenes into the genome [17]. In their endogenous form, autonomous transposons carry the coding sequence for a transposase enzyme, flanked by its recognition sites. The transposase excises the DNA between its recognition sites, and reinserts the DNA at another site in the genome. The *Tol2* transposon is a member of the hAT family, cloned from *Oryzias latipes* [18]. To adapt *Tol2* for use as a gene delivery vehicle, the transposase coding sequence was stripped from the transposon and replaced with a promoter and transgene [19]. mRNA encoding the transposase is provided in *trans*, ensuring that the engineered transposon does not relocate in the genome (Figure 1A). *Tol2*-mediated transgenesis has been achieved in several vertebrate species including teleost fish [20], so we chose to apply this technology to *A. burtoni*.

Here we demonstrate the successful use of *Tol2*-mediated transgenesis in *A. burtoni*, a haplochromine cichlid from Lake Tanganyika thought to be similar to the most recent common ancestor of ∼1500 cichlid species in Lakes Malawi and Victoria [5]. *A. burtoni* is thus well-suited for the study of genes and *cis* regulatory regions that may have influenced the trajectory of this spectacular radiation. In addition to its predicted similarity to the ancestor of the haplochromines in Lakes Malawi and Victoria, *A. burtoni* is also a model organism for behavioral studies [21], [22], [23], [24], [25], [26] and, more recently, the molecular and cellular basis of behavior [27], [28], [29]. This experimental model may be useful for studies of developmental lineage tracing and parentage, and presages the use of transgenic *A. burtoni* in functional studies of evolutionary biology and behavioral neuroscience.

## Results

### Generation of transgenic *A. burtoni* by microinjection

**Figure 2:**
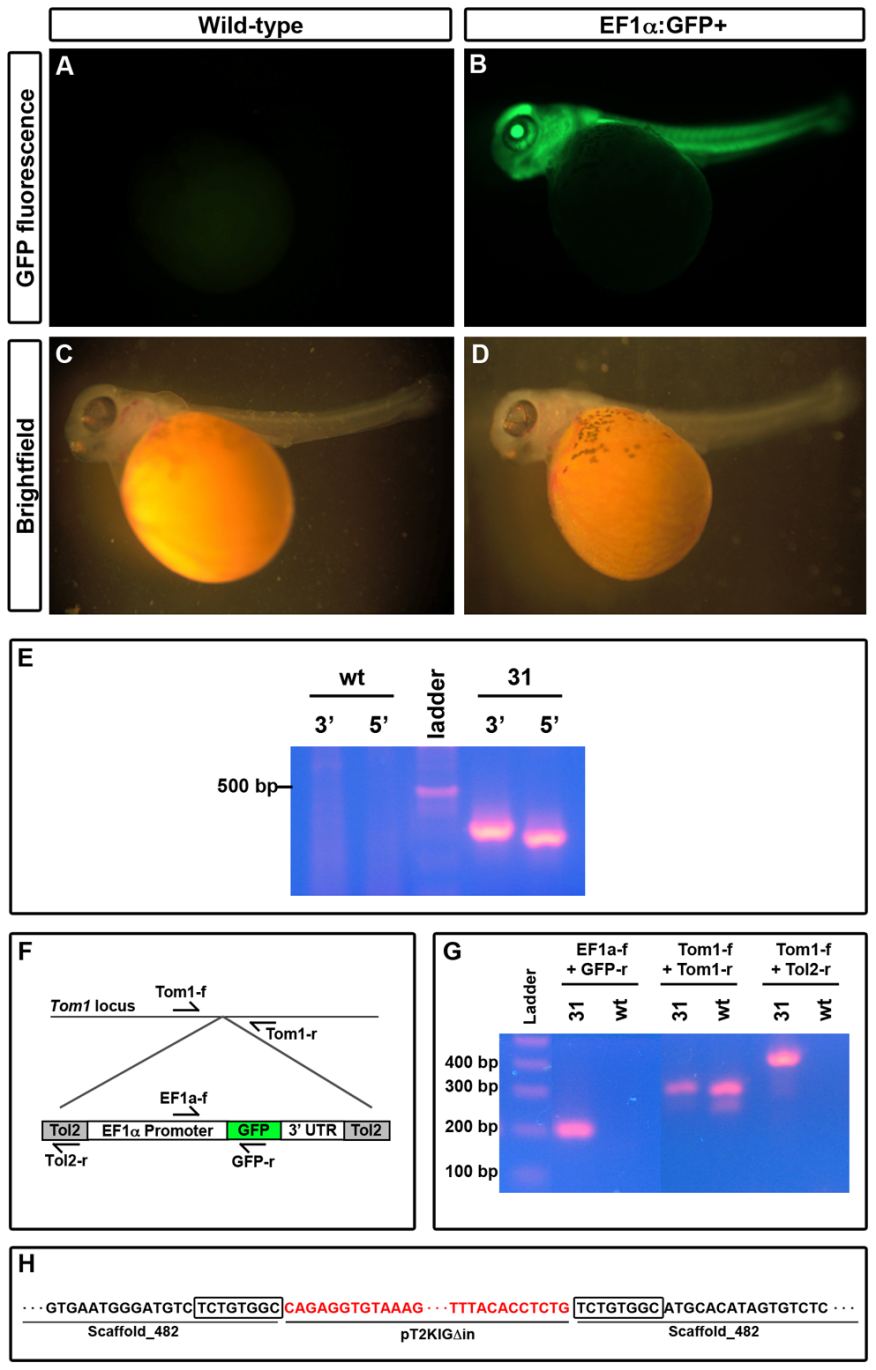
*Tol2* catalyzes transgene integration in germline. Transgenic 5(B), while a wild-type sibling does not (A). Brightfield images of the larvae depicted in A and B are provided for comparison (C, D). E) Splinkerette nested PCR amplifies region surrounding transgene insertion site with primers in the splinkerette adapter (Splink3, Splink6), combined with primers at either the 5′ (Tol2-r, Splink7) or the 3′ (Splink5, Splink8) end of the transgenic construct. F) Schematic of locations for primers in the *Tom1* locus and the integrated *EF1α*:*GFP* cassette. G) PCR from genomic DNA template of line 31 GFP+ fish and wild-type siblings demonstrating *EF1α*:*GFP* integration at the *Tom1* locus. H) Sequencing of PCR products shows that the *EF1α*:*GFP* cassette integrated on scaffold 482. Sequence in red matches the *Tol2* flanking region from pT2KXIG
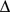
in, while sequence in black matches the *A. burtoni* genome. The boxed sequence is an 8 bp duplication of the genomic locus indicative of *Tol2*-catalyzed integration.

**Figure 3:**
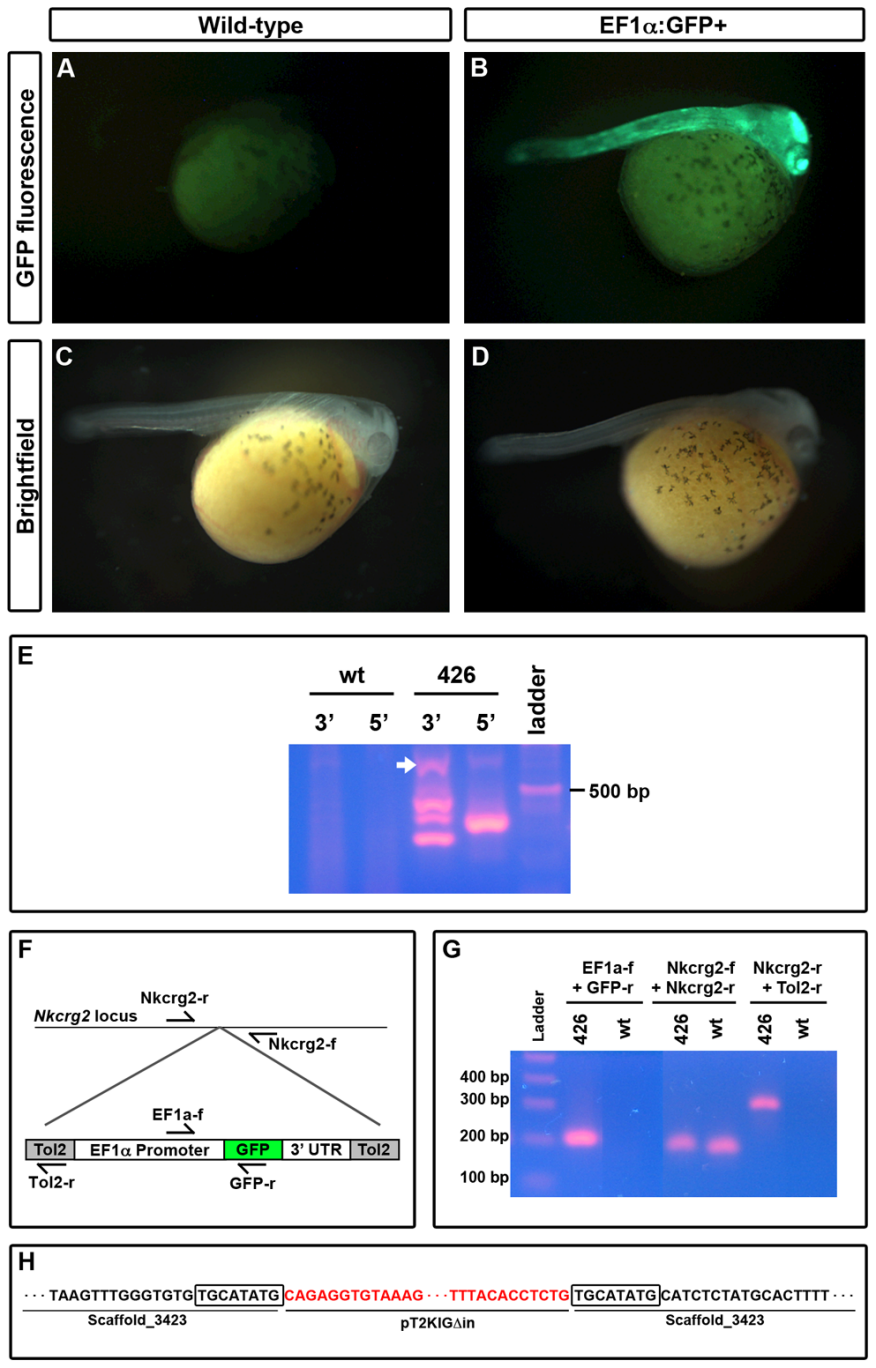
GFP expression and genetic analysis of *EF1α*:*GFP* line 426. Transgenic 4(B), while a wild-type sibling does not (A). Brightfield images of the larvae depicted in A and B are provided for comparison (C, D). E) Splinkerette nested PCR amplifies region surrounding transgene insertion site with primers in the splinkerette adapter (Splink3, Splink6), combined with primers at either the 5′ (Tol2-r, Splink7) or the 3′ (Splink5, Splink8) end of the transgenic construct. All bands were sequenced; arrow indicates band that provided sufficient quality sequence for mapping. F) Schematic of locations for primers in the *Nkcrg2* locus and the integrated *EF1α*:*GFP* cassette. G) PCR from genomic DNA template of line 426 GFP+ fish and wild-type siblings demonstrating *EF1α*:*GFP* integration at the *Nkcrg2* locus. H) Sequencing of PCR products shows that the *EF1α*:*GFP* cassette integrated on scaffold 3423. Sequence in red matches the *Tol2* flanking region from pT2KXIG
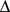
in, while sequence in black matches the *A. burtoni* genome. The boxed sequence is an 8 bp duplication of the genomic locus indicative of *Tol2*-catalyzed integration.

In order to insert transgenic sequences into the genome of *A. burtoni*, we utilized the *Tol2* transposase system. This approach delivers to the single-cell embryo a plasmid containing a promoter and *GFP* transgene, flanked by recognition sites for the *Tol2* transposase (Figure 1A). We injected plasmid carrying GFP coding sequence under the control of the ubiquitous *Xenopus EF1α* promoter (pT2KXIGΔin) [30], along with *Tol2* mRNA into single-cell embryos harvested from mouthbrooding females 30 minutes after spawning. Embryos were monitored for viability for two weeks, and scored for GFP expression. Of the 286 injected embryos, 14% survived until 5 days post-fertilization (dpf). We assessed GFP expression at 4–7 dpf, after embryos had hatched, around the time of jaw extension and gill formation, but before pigmentation obscured underlying tissues [31]. Approximately half of the surviving embryos expressed GFP (19/41 embryos; Figure 1B), however this expression varied across animals from weak and/or sparse labeling (10/41 embryos) to robust labeling (9/41 embryos). The variegated expression patterns may result from mosaic uptake of the transgenic vector across embryonic cells or differing loci of genomic integration, and therefore we chose embryos expressing robust GFP as being most likely to transmit the transgene to offspring. We mated six such fish, and found that two transmitted the transgene to offspring (F1 fish), in lines 31 and 426. A high proportion of offspring from these founders carried the GFP transgene (38% for line 31, n = 13; 27% for line 426, n = 56), indicating extensive population of the germline by transgene-positive cells. In contrast to the mosaic GFP pattern observed in founder fish, GFP expression in F1 fish from line 31 appeared widespread and bright (Figure 2B), as expected for an animal carrying the *EF1α*:*GFP* transgene in all cells. GFP expression in line 426 was more restricted, with bright labelling observed in the brain and laterally, along the length of the body (Figure 3B). Transmission of the *EF1α*:*GFP* transgene from F1 to both male and female F2 fish occurs at a frequency to be expected for a single, autosomal allele (51% GFP+; n = 117 F2 larvae from line 31). The expression patterns for both lines have remained stable for two generations. This finding, in combination with the inheritance pattern, indicates that the *Tol2*-flanked transgenic cassette is not silenced or excised from the *A. burtoni* genome.

### Mapping transgene integration sites

The successful transmission of the transgene from founder to F1 offspring indicates that it integrated into the genome in the founders' germlines. To determine whether insertion was *Tol2*-mediated or due to random integration, we sequenced insertion sites using splinkerette analysis [32]. We digested tailfin genomic DNA, and ligated these fragments to splinkerette adapters, followed by two rounds of nested PCR amplification with primers specific for the transgene and splinkerette. This amplification yielded distinct bands for each transgenic line analyzed (Figures 2E, 3E). Sanger sequencing of the amplicons revealed the genomic sequence surrounding the transgene insertion site (Figures 2H, 3H). Immediately surrounding the *Tol2* recognition sites is a duplication of an 8 bp sequence, a feature indicative of *Tol2* transposition [33]. In addition, the sequence of the integrated construct at the junction with *A. burtoni* genomic DNA sequence matches precisely with that observed in *Tol2*-mediated integrations in zebrafish [34]; random integration would have resulted in the incorporation of larger swathes of the plasmid.

Next we asked whether we could identify the genomic locus of integration in each line. We used BLAST [35] to probe the *A. burtoni* genome (Broad Institute, Boston, MA, USA; v1) with the 80–120 bp sequences surrounding the transgene. This analysis indicated that line 31 carries the transgene on scaffold 482, while line 426 integrated on scaffold 3423. To confirm integration at the predicted locus, we designed primers flanking the putative integration site (outside the region obtained from splinkerette sequencing; Table 1), and performed genomic PCR with a locus-specific primer and a transgene specific primer (Figures 2F-G, 3F-G). Sanger sequencing of the amplicons generated by these specific primers confirmed that line 31 integrated into scaffold 482, in an intron of the *Target of myb 1* (*Tom1*) locus (Figure 2H). Line 426 integrated on scaffold 3423, in a sparsely annotated region with homology to *Natural killer cell receptor gamma 2* (*Nkcrg2*; Figure 3H). These results indicate that the *Tol2* transposase catalyzes the transposition of the *EF1α*:*GFP* transgene from a plasmid into the *A. burtoni* genome, and that the transgene can be expressed throughout the fish.

**Table 1:** Oligonucleotides used.

Primer name	Sequence
Nkcrg2-f	AGT GGT GGA AGG GAA CAA TG
Nkcrg2-r	TTA GCC TGA GCA GCC ACT TC
Tom1-f	AAT ATA CCT CTG TGT CTA CCA G
Tom1-r	AGC ATA ACT AAA TAA GCT GTC AG
EF1a-f	CCT ACA GCT CCT GGG CAA CG
GFP-r	AGC TTG CCG TAG GTG GCA TC
Splink1	TAA CCG TTG CTA GGA GAG ACC GTG GCT GAA TGA GAC TGG TGT CGA CAC TAG TGG CAT G
Splink2	CCA CTA GTG TCG ACA CCA GTC TCT AAT TTT TTT TTT CAA AAA AA
Splink3	AAC CGT TGC TAG GAG AGA CC
Tol2-r	GCG TGT ACT GGC ATT AGA TTG
Splink5	AAT TAA ACT GGG CAT CAG CG
Splink6	GCT GAA TGA GAC TGG TGT CG
Splink7	TAA ATA CAA ACA GTT CTA AAG CAG
Splink8	ATT GGT TTG GTA ATA GCA AGG G

Primers used to detect and confirm transgene insertion are detailed.

### GFP expression in tissues of transgenic fish

**Figure 4:**
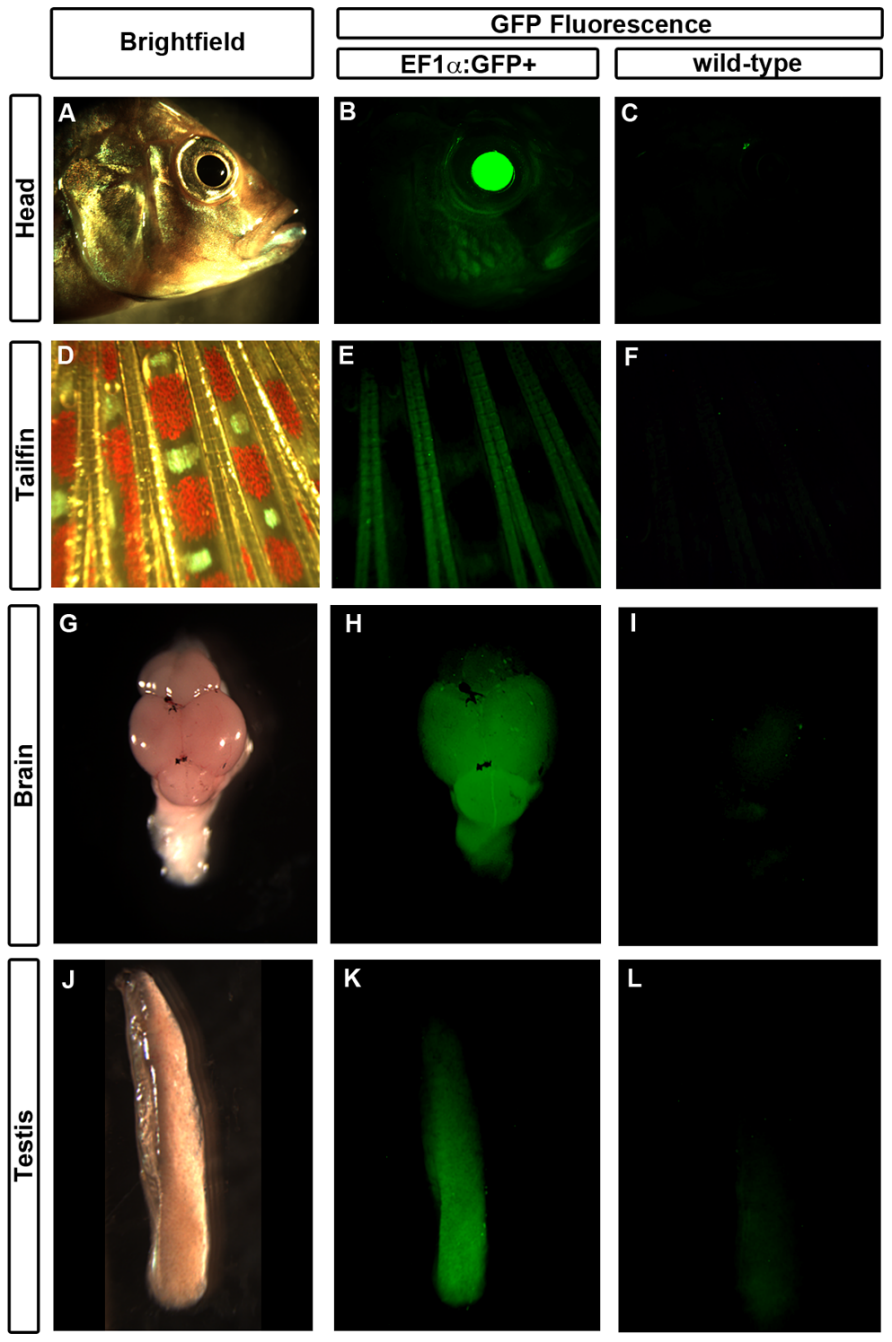
*EF1α*:*GFP* drives widespread expression of fluorescent protein in adult fish. Transgenic fish (B, E, H, K) from line 31 exhibit GFP fluorescence in eye (B), tailfin (E), brain (dorsal view, posterior toward bottom; H), and testis (K). Brightfield (A, D, G, J) and wild-type sibling (C, F, I, L) tissues are provided for comparison.

We sought to determine whether *Tol2* transgenic constructs are capable of expression throughout the body of *A. burtoni*. Observation of widespread GFP expression in larvae of line 31 carrying the transgene suggested that the *EF1α* promoter could drive expression in most tissues. To test this, we dissected adult male *EF1α*:*GFP+* and wild-type siblings (as established by genomic PCR), and focused on tissues with low autofluorescence under a fluorescent dissection microscope. We found that in all tissues examined (eye, brain, testes, liver, and fin), fish carrying the *EF1α*:*GFP* transgene exhibited GFP fluorescence (Figure 4 and data not shown).

## Discussion

We took advantage of the *Tol2* transposon system [20] to introduce the *EF1α*:*GFP* transgene into the genome of *A. burtoni*. We generated two lines that transmitted *EF1α*:*GFP* through the germline and whose progeny exhibit GFP expression. The expression pattern was the same across all animals within a line, indicating that *Tol2* transgenes are stable in *A. burtoni*. One line (31) exhibits strong expression of GFP in most tissues examined (Figure 4). Importantly, these transgenic fish that express the transgene extensively do not exhibit any gross developmental abnormalities, and have body size, fertility, and lifespan similar to their wild-type siblings. Additionally, through video observations of adult transgenic fish we confirmed that they display locomotion and social behaviors typical of wild-type fish (data not shown). The integration site of *EF1α*:*GFP* in this line is a predicted intron of the gene encoding Tom1, a protein about which relatively little is known. It has been suggested to play a role in endosomal trafficking [36], but no animals with loss- or gain-of-function mutations have been studied. We have not observed phenotypes in line 31 consistent with endosome disorders [37]. The animals that we have examined thus far are hemizygous for *EF1α*:*GFP*. Analysis of homozygous fish with disruptions of both *Tom1* alleles may reveal a recessive phenotype that illuminates the function of this gene. The normal developmental trajectory of *EF1α*:*GFP* fish suggests that *Tol2* transgenesis is compatible with cichlid development.

Analysis of GFP expression patterns suggests future uses for transgenic *A. burtoni*. *EF1α*:*GFP* fish could be used in cell transplantation experiments, pending confirmation of GFP expression in specific cell types of interest. Similar transgenic lines with widespread transgene expression have been used in mouse and zebrafish to perform experiments designed to test autonomy of cell signalling or timing of cell development. Although GFP expression appears widespread in line 31 and the *EF1α* promoter is active throughout the body, we cannot conclude that every cell in the body is GFP+. In practice, many promoters reported to be ubiquitous drive transgene expression in a mosaic pattern [38], [39], including *EF1α* [40] This effect of variation in expression pattern has been exploited to generate transgenic lines that allow the analysis of subsets of neurons of interest [41]. In fact, line 426 exhibits GFP expression in a far more restricted pattern than line 31 (Figure 3B, Figure 2B). Visualization of whole 4 dpf embryos from line 426 revealed GFP expression along the body wall, and in a subset of brain regions, including optic tectum. Within the adult brain, GFP expression was restricted to a small population of cells localized to the preoptic hypothalamus, the pallial division of the telencephalon, and the nucleus of the lateral torus (data not shown) [42], [43]. The restricted pattern of expression suggests that transgene expression is modified by local *cis* regulatory elements, and future studies could make use of randomly inserted transgenes with a minimal promoter to “trap” enhancers, allowing the analysis of function of specific subsets of cells [19], [44].

Transgenic fish have previously been generated for species that are genetic model organisms including zebrafish, medaka, stickleback, and killifish [45], [46], [47], [48]. Additionally, transgenic versions of fish that are major food sources have been generated, including salmon, carp, and tilapia [49], [50], [51], [52], [53]. Notably, tilapia (*Oreochromis niloticus*) is a cichlid species as well, although it is a basal-branching species ∼40 million years removed from the most recent common ancestor of the haplochromine radiation in the Rift Valley lakes [54]. Several intrinsic factors have been proposed as having permitted haplochromine diversification, such as a “generalist” jaw morphology, for which some key developmental genes have been identified [14], [55], and sexual dichromatism [54]. Phylogenetic analysis suggests *A. burtoni* is similar to the ancestral haplochromine that gave rise to the ∼1500 haplochromine species in Lakes Malawi and Victoria and is thus well-suited for the study of genes that may have influenced the trajectory of this explosive speciation [5], [54].

In addition to its advantageous position in the cichlid phylogeny, *A. burtoni* is also a model organism for behavioral studies [27] and, more recently, the molecular and cellular basis of behavior [25], [29]. Cichlid transgenesis has the potential to generate animals expressing transgenes of interest in a desired cell population, using cell-type-specific regulatory elements (e.g. promoters). For example, gonadotropin-releasing hormone (GnRH) neurons are critical for reproduction in all vertebrates, but many aspects of their connectivity and function remain untested. *A. burtoni* GnRH neuron morphology and electrophysiology are responsive to social cues [56], [57]; expressing GFP in GnRH neurons would allow fine-scale mapping of neuronal processes and recording of neural activity in a cell-type specific manner. Other transgenes would enable functional tests of the role of defined cell populations through neuronal activation, silencing, or ablation [16]. The transgenic toolbox can also reveal the axons and dendrites of neurons or their pre- and postsynaptic partners, delineating the larger neural circuit in which the cells of interest operate. These capabilities will greatly assist using cichlids as a model for understanding the neural bases of sophisticated social behaviors. The diversity of cichlid species represents a natural mutagenic screen, and the use of transgenesis will allow a dissection of the molecular and cellular basis of the phenotypes generated during cichlid evolution.

## Methods

### Animals

All animals were handled in strict accordance with a protocol approved by Stanford University's Administrative Panel on Laboratory Animal Care (Protocol Number: 9882). *A. burtoni* were housed in 60 liter aquaria under conditions designed to mimic their natural environment [21]. Aquarium water with cichlid salt and Tanganyika buffer (Seachem, Madison, GA) was 28°C and pH 8, and full-spectrum lights illuminated the tanks in a 12-hour light:12-hour dark cycle. Tank bottoms were covered with gravel, and males were provided with half a clay flowerpot in which they established a territory and spawned with gravid females. Fish were fed each morning with cichlid flakes and pellets (AquaDine, Healdsburg, CA), and females also received brine shrimp (San Francisco Bay Brand, Newark, CA). Fish were housed separately by sex to control the timing of spawning; males were housed singly, while females were housed in groups of ∼20 fish.

On the day of spawning, ∼5 females were transferred from single-sex housing into a male's tank, and interactions were monitored for spawning. *A. burtoni* females are mouthbrooders; a female that has spawned is readily identified by her distended, opaque buccal cavity. If a successful spawning was observed within 15 minutes, the fish were given an additional 30 minutes to fertilize eggs before the brood was removed from the female's mouth. While awaiting injection, fertilized eggs were gently agitated in tank water at room temperature (25°C) containing 1 mg/L methylene blue (Sigma) to inhibit fungal growth.

### Transgenic *A. burtoni* production

*Tol2*-mediated transgenesis was achieved by co-injection of a solution containing 25 ng/µL of the pT2KXIGΔin plasmid containing the *EF1α*:*GFP* cassette flanked by *Tol2* recognition sites [30], 25 ng/µL of *Tol2* transposase mRNA, and 0.5% Texas Red-conjugated dextran for visualization of injected solution (3000 MW; Life Sciences). pT2KXIGΔin carries the *EF1α* promoter, driving expression of enhanced *GFP*. Expression levels are enhanced by the inclusion of the rabbit *beta-globin* intron and SV40 poly(A) 3′ UTR. *Tol2* transposase mRNA was transcribed *in vitro* from the plasmid pCS-TP [19] using SP6 polymerase (Ambion mMessage kit) according to manufacturer's instructions.

For injection, embryos were held in place between the edge of a glass-walled slide chamber and a notched coverslip similar to Hosemann et al., 2004 [45]. Injection needles were produced by pulling borosilicate capillary tubes (1.0 mm O.D., 0.58 mm I.D.; Harvard Apparatus) with a Sutter P-97 micropipette puller (heat, 482; pressure, 450; pull, 90; velocity, 45; time, 200). Injections were performed under a stereomicroscope (Zeiss Stemi SV6). Using a micromanipulator, needles were inserted into the single cell of the embryo, and a Picospritzer II (General Valve Corporation) delivered ∼1 nL at 40 psi of solution containing the pT2KXIGΔin and *Tol2* transposase mRNA. Injections continued until two-cell embryos were observed, around 1.5 hours post-fertilization.

Following injection, the developing embryos were gently tumbled in a modified bubbler submersed in an aquarium containing 1 µg/mL methylene blue. Injected embryos were monitored daily, and any dead embryos were removed immediately. We assessed GFP expression at 4–7 dpf, after embryos had hatched and developed beating hearts, around the time of gill development (∼stage 17, according to [31]). We detected GFP using a dissecting stereomicroscope equipped with a fluorescent light source (Leica MZFLIII with GFP2 filter; excitation 480/40 nm, barrier 510 nm). Embryos were qualitatively assigned as expressing no GFP, low and/or sparse GFP (<25% of embryo labelled), or high, robust GFP (>25% of embryo labelled). Those embryos with extensive GFP expression were selected for breeding. When free-swimming larvae had completely absorbed their yolk sacs, around 14 dpf, they were group-housed in aquaria. Upon reaching sexual maturity, as judged in males by the display of bright, adult male-typical coloration (typically ∼10 weeks post-fertilization) [58], fish expressing robust GFP were co-housed with wild-type opposite-sex partners from the lab stock, and females were monitored for fertilized broods of eggs. All F1 and F2 fish analyzed were therefore hemizygous carriers of *EF1α*:*GFP*. For adult tissue imaging, animals were anaesthetized with MS-222 (0.175%, Argent Labs, Redmond, WA), rapidly dissected, and imaged with a fluorescent stereomicroscope as above. All images were captured using a SPOT Insight Camera and SPOT Advanced Software (SPOT Imaging Solutions).

### Splinkerette

Splinkerette ligation was performed as described by Horn et al. [32], with modifications [59]. Genomic DNA was purified from dorsal fin clips using DNEasy columns (Qiagen). Genomic DNA was digested for three hours with Nla III (NEB). Splinkerette adapters were generated by annealing two synthetic oligonucleotides, Splink1 and Splink2 (Table 1). Nla III-digested genomic DNA was ligated to the splinkerette adapter at 16°C overnight, and then the reaction was column purified (Qiagen) and eluted in 30 µL. 1 µL of the ligation product was used as template in PCR with the primer Splink3 combined with Tol2-r (5′ lane in Figure 2E and Figure 3E) or Splink5 (3′). 1/100^th^ of the PCR reaction was run in a second round with the primers Splink6 combined with Splink7 (5′) or Splink8 (3′). This PCR reaction was run on a 2% agarose gel, and bands were excised, gel purified (Qiagen), and analyzed by Sanger sequencing (ElimBio, Hayward, CA).
